# Gene polymorphisms as susceptibility factors in Brazilian asthmatic children and adolescents

**DOI:** 10.1186/1939-4551-8-S1-A235

**Published:** 2015-04-08

**Authors:** Isabel Rugue Genov, Angela Falcai, Alessandra Pontillo, Antonio Condino Neto, Marcia Carvalho Mallozi, Dirceu Sole

**Affiliations:** 1 Federal University of São Paulo, Brazil; 2 Universidade De São Paulo, Brazil; 3 Brazilian Society, Brazil

## Background

Asthma is a complex disease due to the contribution of both genetic and environmental factors. Several genes and polymorphisms have been associated to asthma susceptibility and development, leading to distinct clinical patterns. The aim of this study was to analyze sixteen genetic polymorphisms in eleven genes previously associated to asthma in a Brazilian family-based population study.

## Methods

Sixteen single nucleotide polymorphisms (SNPs) in *TNF*, *IL6*, *IFNG*, *TGFB1*, *IL10*, *CD14*, *TLR4*, *TLR7*, *TLR8*, *FLG*, *ADRB2*genes were genotyped in 311 family trios (n=944), by SSP-PCR or allelic-specific Taqman assay techniques. PLINK and Haploview softwares were used for data analysis.

## Results

TDT analysis showed that, among the 16 SNPs studied, three SNPs were associated to susceptibility to the development of asthma, rs1800629 (*TNF*-308) minor A allele (*p*=0,0031; OR=0,5), rs1800795 (IL6-174) minor allele C (*p*=1,87exp-7; OR=0,35) and rs1800471 (*TGFB1*+915) minor allele C (*p*=1,34exp-8; OR=0,11) were significantly less frequently transmitted within the families, suggesting a protective effect of these alleles against the development of asthma. After ethnicity stratification, the same SNPs showed significant association in White patients (n=168), but not in Mullato patients (n=154) for *TNF-308A*, which is possibly related to number of patients analyzed in this population. *IL6*-174, *TNF*-308 and *TGFB1*+915 haplotype association analysis showed risk to asthma (GGG, OR=5,3, p=1E-5) and protection to asthma (CGC, OR=0,24, p=1,9E-4).

## Conclusions

Our results revealed a protective association of *TNF*-308A, *IL6*-174C e *TGFB1*+915C variants in a Brazilian family-based association study confirming previously reported data and established two new haplotypes conferring asthma susceptibility.

